# Retraction Note: Hypoxia impairs decitabine-induced expression of HLA-DR in acute myeloid leukaemia cell lines

**DOI:** 10.1186/s13148-026-02074-4

**Published:** 2026-02-09

**Authors:** Sam Humphries, Sean M. Burnard, Courtney D. Eggins, Simon Keely, Danielle R. Bond, Heather J. Lee

**Affiliations:** 1 School of Biomedical Sciences and Pharmacy, The University of Newcastle, Callaghan, NSW 2308 Australia; 2 Precision Medicine Research Program, Hunter Medical Research Institute, New Lambton Heights, NSW 2305 Australia; 3 Immune Health Research Program, Hunter Medical Research Institute, New Lambton Heights, NSW 2305 Australia

**Retraction Note to: Clinical Epigenetics (2025) 17:8** 10.1186/s13148-025-01812-4

The authors have retracted this article. Following publication, a mistake related to cell line identity was discovered. Data attributed to MV-4-11 cells in the original publication was obtained from mislabelled MOLM-13 cells. Data from HL-60 and MOLM-13 cells are unaffected by this mistake. While hypoxia impaired decitabine-induced expression of HLA-DR in MOLM-13 cells, the result has not been confirmed in MV-4-11 cells.

All authors agree with this retraction.

